# Self-Folding of Naked Segment 8 Genomic RNA of Influenza A Virus

**DOI:** 10.1371/journal.pone.0148281

**Published:** 2016-02-05

**Authors:** Elzbieta Lenartowicz, Julita Kesy, Agnieszka Ruszkowska, Marta Soszynska-Jozwiak, Paula Michalak, Walter N. Moss, Douglas H. Turner, Ryszard Kierzek, Elzbieta Kierzek

**Affiliations:** 1 Institute of Bioorganic Chemistry, Polish Academy of Sciences, Noskowskiego 12/14, 61–704 Poznan, Poland; 2 Department of Chemistry, University of Rochester, Rochester, New York, 14627, United States of America; Washington University School of Medicine, UNITED STATES

## Abstract

Influenza A is a negative sense RNA virus that kills hundreds of thousands of humans each year. Base pairing in RNA is very favorable, but possibilities for RNA secondary structure of the influenza genomic RNA have not been investigated. This work presents the first experimentally-derived exploration of potential secondary structure in an influenza A naked (protein-free) genomic segment. Favorable folding regions are revealed by *in vitro* chemical structure mapping, thermodynamics, bioinformatics, and binding to isoenergetic microarrays of an entire natural sequence of the 875 nt segment 8 vRNA and of a smaller fragment. Segment 8 has thermodynamically stable and evolutionarily conserved RNA structure and encodes essential viral proteins NEP and NS1. This suggests that vRNA self-folding may generate helixes and loops that are important at one or more stages of the influenza life cycle.

## Introduction

Influenza A virus causes yearly epidemics that kill hundreds of thousands of humans [1] and undergoes genetic reassortments, that produce infrequent, but more deadly pandemics [2]. The virus is classified by subtypes differing in the viral surface proteins, hemagglutinin (HA) and neuraminidase (NA). Influenza is a negative sense (-)RNA virus with a segmented genome. Its life cycle involves RNA exclusively, no DNA intermediate is involved [3, 4]. The genomic RNA orchestrates various functions including replication, transcription, translation, and virion assembly. Despite influenza genomic RNA importance, knowledge of its structure is limited. Genomic RNAs (vRNA) share a common organization consisting of a central open reading frame (antisense) flanked on both ends by short (19–58 nt) untranslated regions (UTRs) [5]. The base paired 5' and 3' ends form a structure called *panhandle* that is recognized by the viral polymerase complex [6]. Binding of polymerase induces a partial conformational change in the RNA structure [7, 8], thought to allow promoter activity and synthesis of viral mRNA utilizing an unusual host “cap-snatching” endonuclease activity [9]. Although vRNA UTR structures and roles are reasonably well defined [10], potential structural motifs in the vRNA and also in the template (cRNA) for RNA dependent polymerase synthesis of vRNA are not known.

There is evidence for a few other sequences and structures in influenza vRNA that play essential roles in the virus life cycle [11, 12]. For example, gel retardation and cell culture experiments have identified interactions important in packaging [12–16]. Functional importance of a predicted pseudoknot structure in the segment 5 vRNA packaging region was confirmed by plaque assays of mutant viruses with disrupted structure and with folding restored by compensatory substitutions [12]. Moreover, ADAR1 editing [17] of vRNA was observed in segments encoding M1 [18] and HA [19] proteins. ADAR1 activity is a restriction factor important for influenza virus replication [20] and is specific to helical regions of RNA. These findings support the idea of RNA structure in influenza vRNA. In general, viral genomes are thought to contain RNA structure, but few details are currently known [21].

The potential for additional stably folded RNA secondary structures has been identified in influenza A through sequence analysis of both (+) and (-) sense RNA [22]. Prediction of thermodynamically stable secondary structure combined with suppression of synonymous codon usage (SSCU) sequence comparison predicted at least twenty conserved local secondary structures [23], two of which were previously predicted [11, 24]. A combination of in vitro chemical and isoenergetic microarray mapping validated five mRNA regions as containing stable structure [25–29].

Much of the time, vRNA of influenza A is associated with the heterotrimeric viral polymerase and multiple copies of the viral NP protein in a ribonucleoprotein complex (vRNP) [30, 31]. This vRNP is packaged into active virions. Results from cryo EM and from *in vitro* mapping studies of RNP particles isolated from virus [32, 33] suggest that NP protein binding destabilizes folded viral RNA structure [4, 30]. Several studies, however, indicate that vRNA is not covered by NP in such a way to prevent all regions from folding [34–36]. For example, influenza A infected cells typically have ~ 24 nucleotides per NP and the average periodicity of NP on the genomic RNA is 32 ribonucleotides [4]. Salmon anemia virus NP, which is very similar to influenza A NP, however, only binds 12 nucleotides of a single stranded RNA [35]. This suggests roughly half to a third of the RNA is not bound to NP. Cryo EM studies also show that the RNA binding sites on NP leave large sections of RNA exposed, which explains the susceptibility of influenza virus RNP to ribonucleases [4, 30]. Moreover, cryo EM indicates that "the RNA sequences that are most intimately bound by NP are not directly accessible for transcription or replication” suggesting “at least local disassembly of the RNP is required” [4].

During active infections of cells, virions are broken down, and vRNPs are dynamic entities where regions of protein-free (“naked”) vRNA may be present at various stages (e.g. in newly synthesized regions of vRNAs and during synthesis of mRNA and cRNA). RNA-RNA interactions are quite strong, e.g. a single GC pair can stabilize a helix by 3 kcal/mol at 37°C [37]. Moreover, hairpins can fold within tens of microseconds [38]. Thus, local structure can form quickly and be thermodynamically stable.

Oligonucleotides can affect RNA function in influenza [13, 39, 40]. Presumably, oligonucleotide sequence dependent effectiveness depends on target structure. Thus insights into RNA structure can facilitate design of oligonucleotides that can be agents to reveal structure-function relationships, and potential lead therapeutics.

In this work, the *in vitro* base pairing of the protein-free entire segment 8 vRNA (vRNA8, strain A/Vietnam/1203/2004 (H5N1)) is modeled on the basis of chemical mapping combined with predicted thermodynamics and sequence/structure comparison. Additionally, microarray mapping is consistent with the *in vitro* deduced pairing. H5N1 virus subtype was used as a model strain to find thermodynamically stable and evolutionarily preserved RNA secondary structure motifs. This strain was isolated from humans. It has high medical importance because it is one of a few avian influenza viruses found in humans and causes high morbidity and mortality upon infection [41]. H5N1 virus subtype has a high potential of causing a pandemic if it acquires ability to transmit easily among humans [42].

Results presented here show that naked H5N1 vRNA8 has structural motifs *in vitro* that are thermodynamically stable and conserved throughout influenza A strains. Much of the folding is local, so individual motifs may only form at particular times of the viral life cycle. This information can facilitate design of nucleic acid—based and/or small organic molecules that could be used in cell culture to provide insight into functional significance and/or as therapeutics.

## Materials and Methods

### Materials

Standard phosphoramidites for oligonucleotide synthesis (DNA, RNA, 2'-O-methyl RNA), 6-FAM phosphoramidite and C6-aminolinker were purchased from Glen Research. Phosphoramidites of LNA, LNA 2,6-diaminopurine riboside and 2'-O-methyl-2,6-diaminopurine riboside were synthesized according to published procedures [43]. Dimethyl sulfate (DMS) was from Aldrich and *N*-methylisatoic anhydride (NMIA) was from Molecular Probes. Reverse transcriptase SuperScript III was from Invitrogen. AmpliScribe T7 Transcription Kit and RNase H were from Epicenter. Pfu polymerase and dNTP were from Fermentas. Roche was provider of ddNTP. Restriction enzymes: EcoRI and PstI were from Promega. DH5α competent cells, agarose and HybriSlip hybridization cover were bought from Invitrogen. T4 polynucleotide kinase was product of EURx. Silanized slides were purchased from Sigma.

### Chemical synthesis of oligonucleotides

DNA and modified DNA-LNA primers for PCR and reverse transcription, and 2’-O-methyl-LNA modified probes were synthesized by the phosphoramidite approach on a MerMade synthesizer. Oligonucleotide primers for reverse transcription were synthesized with fluorescein on the 5’ end (6-FAM). Oligonucleotide probes have a C6-aminolinker on the 5' end. Synthesized oligonucleotides were deprotected and purified according to published procedures [37, 44], and their molecular weights were confirmed by mass spectrometry (MALDI-MS). Concentrations of all oligonucleotides were measured with a UV spectrophotometer (Picodrop-Syngen).

### RNA synthesis

RNAs were synthesized by *in vitro* transcriptions. DNA template for vRNA8 (875 nt) was obtained by PCR from vector pPol1 using appropriate primers (Table A in S1 File). The pPol1 vector containing DNA of segment 8 influenza strain A/Vietnam/1203/2004 (H5N1) was received from Prof. Baek Kim, University of Rochester. The vRNA8 was purified using RNeasy MiniElute Cleanup Kit from Qiagen.

The pPol1 vector was also used to make a plasmid coding for an RNA (mini-vRNA8) devoid of a central 504 nt of segment 8 but retaining the nucleotides required to give optimal packaging of a segment 8 encoding GFP protein [45]. Firstly, two fragments (182 nt from 5’-end and 189 nt from 3’-end) were amplified. The primers used to copy the first fragment included restriction sites for EcoR1 and BamH1 enzymes, respectively, from the 5’ and 3’end. Similarly, the second fragment contains restriction sites for BamH1 and Pst1 enzymes (Table B in S1 File). Digestion with BamH1 enzyme was conducted according to Promega protocol. After ligation of both fragments at 4°C for 16 h, the second polymerase chain reaction was run with primers containing restriction sites for EcoR1 and Pst1 enzymes (Table B in S1 File). The amplification product was cloned into pUC19 vector. From the obtained vector, the DNA template of mini-vRNA8 was amplified (primers: Table A in S1 File) and used for transcription of RNA. The mini-vRNA8 contains 182 and 189 nts, respectively, from the 5’ and 3’ ends of vRNA8 and five additional nucleotides (5’GGAUC) corresponding to the BamH1 restriction site giving a total of 376 nts. The RNA was purified by denaturing polyacrylamide gel electrophoresis.

### Chemical modification

As described below, the SHAPE method with NMIA was used to modify flexible riboses [46]. DMS was used to modify Watson-Crick faces of bases in adenosines and cytidines not in Watson-Crick pairs flanked by Watson-Crick pairs. Prior to chemical mapping, RNA was annealed for 5 min at 65°C in folding buffer A (300 mM NaCl, 5 mM MgCl_2_, 50 mM HEPES, pH 7.5) and slowly cooled to room temperature. After folding, the RNAs formed one band on native agarose gels consistent with formation of one structure (Fig A in S2 File). For chemical modification, 1 pmol of RNA per primer was used for readout by reverse transcription. For example, to 27 μl containing 6 pmol of RNA, 3 μl of 40 mM NMIA in DMSO was added. A control reaction was prepared equivalently with 3 μl of pure DMSO instead of NMIA solution. Samples were incubated for 3.5 h at 23°C. For modification with DMS, 3 μl of 300 mM DMS in ethanol was added to 27 μl containing 6 pmol of RNA. A control reaction was prepared equivalently with 3 μl of water instead of DMS solution. Samples were incubated for 15 min at 23°C. Reactions were stopped by ethanol precipitation. A concentration range of chemicals was tested to find conditions where the vRNA would be modified on average once per 300 nucleotides. Final concentrations of 4 mM NMIA and 30 mM DMS were selected.

### Primer extension

Modification sites were identified by primer extension using 6-FAM labeled primers (Table C in S1 File) specific for studied RNA. Six primers were used for vRNA8 and three primers for mini-vRNA8. Two of six DNA primers contained LNA modified nucleotides. The LNA nucleotides increase stabilities of RNA-DNA duplexes and therefore such modified primers increase reverse transcription efficiency for structured regions of RNA [47]. Also, primers could be shorter and still efficiently bind the RNA. The shorter primers allow reading out chemical modification of more nucleotides.

For each reaction, 1 pmol of RNA and 1 pmol of appropriate primer were used. Primer extension reactions were performed at 55°C with reverse transcriptase SuperScript III and the buffer and protocol of Invitrogen. Reactions were stopped by ethanol precipitation. For each primer, two ddNTP ladders were prepared: (most often ddGTP and ddATP). DNA products were separated by capillary electrophoresis using ABI 3130xl Genetic Sequencer (Laboratory of Molecular Biology Techniques at Adam Mickiewicz University in Poznan).

### Processing of chemical mapping data

Results were analyzed with PeakScaner 1.0 program (available from Applied Biosystems). Reactive nucleotides were identified by comparison to dideoxy sequencing ladders along with mass marker. SHAPE intensities at each individual nucleotide were examined manually to identify positions where high background was present in the control experiment. Less than 5% of positions fell into this category and were marked as containing no data. Quantitative SHAPE reactivities for individual datasets were normalized to a scale in which 0 indicated an unreactive site and the average intensity at highly reactive sites was set to 1.0. The normalization factor for each dataset was determined by first excluding the most-reactive 2% of peak intensities and then calculating the average for the next 8% of peak intensities. All reactivities were then divided by this average. This normalization procedure places all reactivities on a scale of 0 to approximately 1.5. In this scale, reactivities >0.650 are considered as strong, 0.250–0.650 as medium and <0.250 as weak, but all calculated reactivities were used for prediction of secondary structure. Nucleotides with no data were indicated as -999. Normalized SHAPE reactivities from each primer extension reaction were processed independently. DMS modification analysis was equivalent to that described above. For both SHAPE and DMS, at least three datasets were obtained from each primer and the average of results was used in the RNAstructure5.3 program [48, 49] for prediction of secondary structure (S1 Dataset).

### Sequence analysis and *in silico* RNA folding

All non-redundant full-length segment 8 sequences of Influenza A virus were obtained from the NCBI Influenza Virus Resource [50]. Ambiguous nucleotides were filtered out leaving a set of more than 14 000 sequences. The reverse complement of these (the vRNA sequences; generated by BioEdit [51]) were folded *in silico* using RNAfold [52, 53] with default parameters. Primary sequences were aligned using MAFFT (FFT-NS-1 strategy) [54]. Minimum free energy secondary structures predicted with RNAfold were mapped onto the primary sequence alignment and consensus helixes (>50% conserved) were identified. These frequently-predicted helixes were used to constrain base pairing in subsequent modeling.

### Prediction of secondary structure using chemical mapping results

RNAstructure [48] uses thermodynamic parameters [49, 55] and free energy minimization for prediction of base pairing [56]. SHAPE reactivities were converted to pseudo free-energy change terms to restrain predictions. For this purpose the text file with normalized SHAPE reactivity (as described above) was input to RNAstructure5.3 using “Read SHAPE reactivity—pseudo free energy” mode with slope 2.6 and intercept -0.8. DMS mapping data were also introduced at the same time, using “chemical modification” mode to apply only strong DMS modifications as constraints. Restricting DMS constraints to strong modification limits potential misinterpretation if there is an ensemble of secondary structures [57, 58].

### Preparation of isoenergetic microarrays

Semi-universal microarrays [59–62] built with isoenergetic probes were synthesized with probes complementary to all studied RNAs. Probes were 2′-O-methyl oligonucleotide pentamers and hexamers with incorporated LNA nucleotides and 2,6-diaminopurine riboside (LNA or 2′-O-methylated nucleotide) [43, 63, 64]. Also several modified heptamers complementary to A/U rich fragments of vRNA8 were synthesized, with 3’-terminal pyrene (Table D in S1 File). Neither RNA target, however, bound to a heptamer.

Specific and negative control probes were printed on the microarray. Each probe was spotted in triplicate, with a spot distance of 750 μm. UUUUU, U, and spotting buffer were used as negative controls. Microarrays were prepared according to the method described earlier [65, 66]. Silanized slides were coated with 2% agarose activated by NaIO_4_.

Microarrays were printed in the European Center of Bioinformatics and Genomics in Poznan, Poland with NanoPrint microarray printer (Arrayit). Printed microarrays were incubated for 12 h at 37°C in 50% humidity. The remaining aldehyde groups on microarrays were reduced with 35 mM NaBH_4_ solution in phosphate-buffered saline solution and ethanol (3:1 v/v). Then slides were washed in water at room temperature (3x), in 1% SDS solution at 55°C, and finally in water at room temperature (3x) and dried at room temperature.

### Hybridization conditions

For hybridization on microarrays, RNA was radioactively labeled from the 5’ end using [γ-^32^P]ATP and T4 kinase. RNA was purified on a 6% polyacrylamide denaturing gel and the radioactivity was measured using scintillation counter MicroBeta (PerkinElmer). Before hybridization, pure labeled RNA was folded as for chemical mapping (65°C for 5 min and slowly cooling to room temperature) in buffer A (300 mM NaCl, 5 mM MgCl_2_, 50 mM HEPES, pH 7.5) or B (1 M NaCl, 5 mM MgCl_2_, 50 mM HEPES, pH 7.5) or C (300 mM KCl, 5 mM MgCl_2_, 50 mM HEPES, pH 7.5) or D (1 M KCl, 5 mM MgCl_2_, 50 mM HEPES, pH 7.5). The RNAs in these conditions formed one band on native agarose gels, consistent with formation of one structure (Fig A in S2 File).

For each microarray, 250 μl of buffer solution with 200 000 cpm (ca. 10 nM) of radioactively labeled RNA was used similar as described earlier [59, 60, 67]. Hybridization took 18 h at selected temperature (4°C, 23°C, or 37°C) in 100% humidity. After hybridization, microarrays were washed 5 min in buffer with the same composition and temperature as for hybridization, then dried by centrifugation (2 min, 2000 rpm). Hybridization was visualized by exposure to a phosphorimager screen and scanning on Fuji Phosphorimager. ImageQuant 5.2 program was used for quantitative analysis. Bindings were normalized to the strongest intensity and have values in range 1–0, marked as: 0.33 ≤ strong, 0.11 ≤ medium <0.33, and no binding <0.11. Experiments were repeated at least three times, and the average of the data is presented. Binding sites of probes are denoted by the middle nucleotide of the complementary RNA region.

### RNase H assays

As for chemical mapping, vRNA8 (6 pmol) was annealed for 5 min at 65°C in folding buffer A and slowly cooled to room temperature. Then, 3 pmol of DNA oligonucleotide (Table E in S1 File) in buffer with DTT (final concentration 1 mM) and 5 units of RNase H were added to final volume 20 μl. A control reaction was prepared equivalently but without DNA oligomer. Reactions were incubated for 30 min at 37°C and then RNase H was inactivated by incubation for 10 min at 65°C. Samples were precipitated with ethanol and cleavage sites were identified with primer extension with six primers specific for vRNA8 (Table C in S1 File).

## Results

### Secondary structure model for naked vRNA8

**Fig 1:**
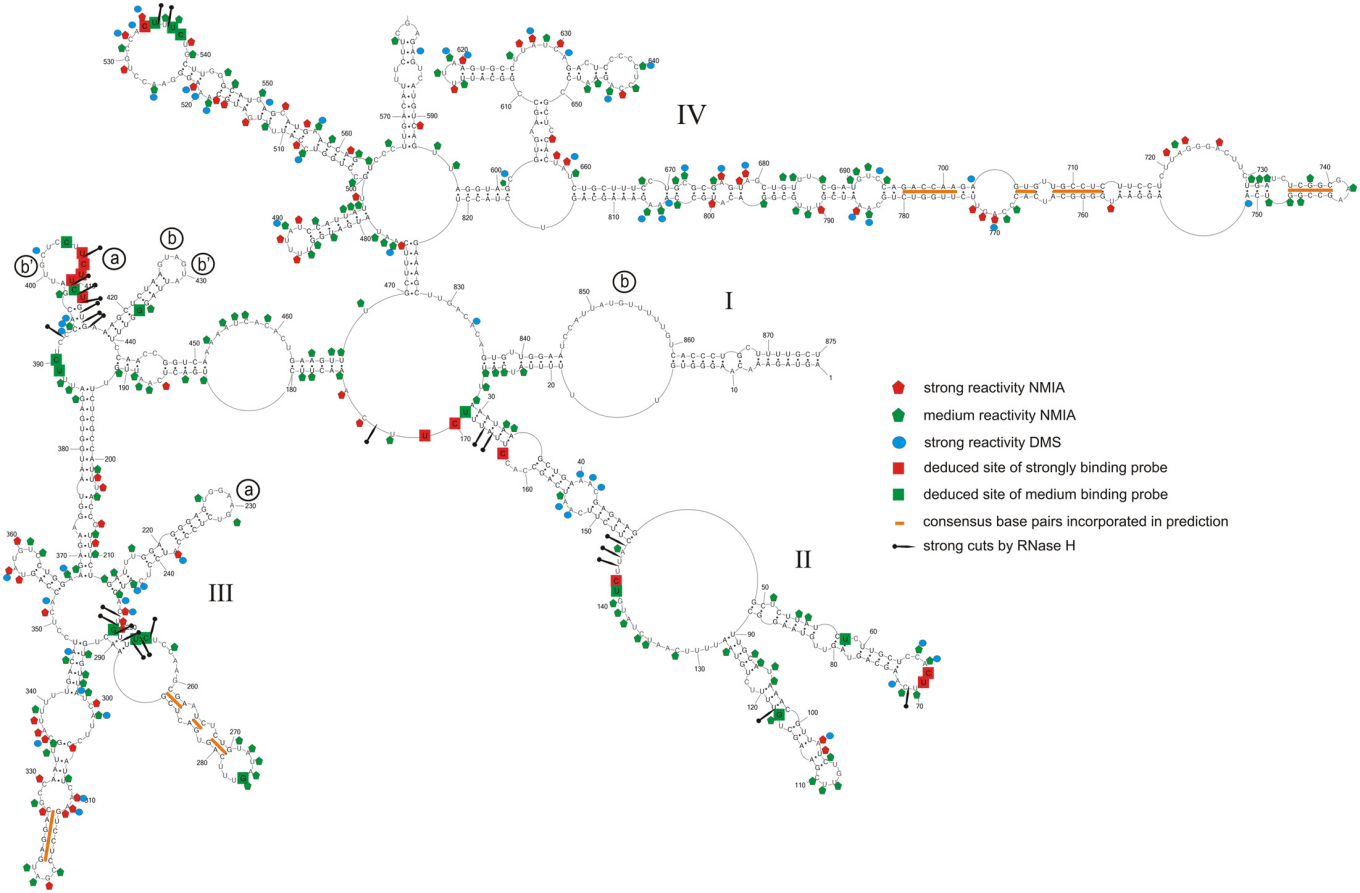
Self-folding of vRNA8 predicted by RNAstructure 5.3 using as constraints: strong reactivity of DMS; consensus base pairs from sequence and structure analysis (orange bars); SHAPE reactivities converted to pseudo- free energies. Additionally there are marked results from microarray mapping in buffer A (300 mM NaCl, 5 mM MgCl_2_, 50 mM HEPES, pH 7.5) at 37°C and also from RNase H cleavage in the same buffer and temperature. Binding sites of probes are denoted by the middle nucleotide of the five nucleotides complementary in the RNA. Possible regions of tertiary interactions are marked by letters in open circles—the same letter marks one interaction (see text). Regions with no read-out by chemical mapping are: 807–875 (NMIA) and 835–875 (DMS). The numbering of vRNA8 is from its 5’ end. The template for the AUG start codon is nucleotides 849–847.

All influenza A segment 8 vRNA sequences were folded *in silico*. This initial analysis revealed five stem regions that are predicted to form across all strains: nucleotides 261-270/277-288, 312-317/322-327, 696-701/775-780, 704-713/758-767 and 736-740/744-748 (the numbering of vRNA8 is from its 5’ end) (Fig 1). These helixes have base pairing conservation of 97.2, 94.9, 85.2, 97.5, and 92.6%, respectively (S2 Dataset).

The secondary structure of naked vRNA8 was chemically mapped in buffer A (300 mM NaCl, 5 mM MgCl_2_, 50 mM HEPES, pH 7.5) at 23°C. NMIA reacted strongly and moderately, respectively, with 65 and 166 of 875 nucleotides. DMS reacted strongly and moderately with 58 and 115 nucleotides, respectively (Fig 1, and S1 Dataset). In total, 60% of nucleotides showed only weak or no reactivity, indicating that vRNA8 is highly structured *in vitro*. Similar reactivity has been observed for 16S rRNA [68].

The data from chemical mapping were input into the RNAstructure5.3 program as described in Material and methods. The mapping data were consistent with the six conserved stem regions, which thus were added as base pairing constraints. The resulting base pairing model of vRNA8 is presented in Fig 1. While not used in modeling, alignment of over 14 000 non-redundant sequences (S2 Dataset) revealed compensating changes for base pairs U60/G77, C63/G74, C99/G117, C661/G814, C664/G811, U662/A808, C694/G782, A697/U779, and A703/U773. The primer complementary to region 769–789 of vRNA8 did not undergo extension with reverse transcriptase, so probably did not hybridize, which is consistent with the strong secondary structure predicted in this region. In the absence of experimental data, RNAstructure predicted 57% of the same base pairs in Fig 1 (see folding in Fig B in S2 File).

The base pairs modeled for vRNA8 in Fig 1 fall into four domains. Domain I (1-27/875-836) has the 5’ and 3’ ends of vRNA8 partially paired, corresponding to the *panhandle* structure previously proposed [5, 6, 10]. The entire vRNA8 structure contains 269 canonical base pairs (including G-U), so 61% of nucleotides are paired. The results imply that naked vRNA8 is highly structured *in vitro*. While there may be an ensemble of structures *in vitro*, the RNA can fold stable secondary structure locally as well as in long distance. For example, the predicted free energies of the hairpins formed by nucleotides 213–246 and 687–789 are, respectively, -13 and -42 kcal/mol, which translate to equilibrium constants for folding of 1X10^9^ and 4X10^29^, respectively. The results suggest that some of the structural motifs can also form *in vivo*.

**Fig 2:**
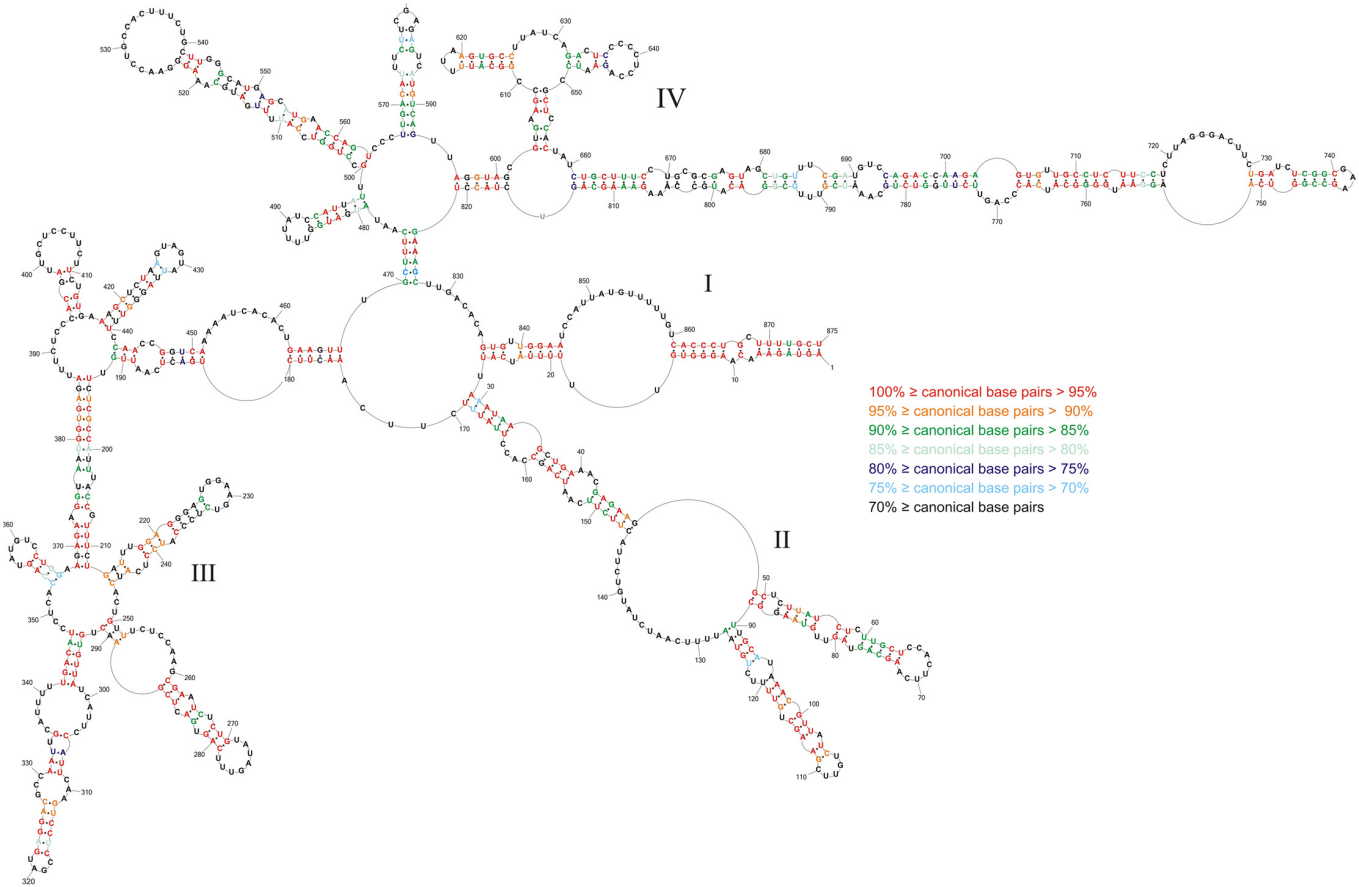
Conservation of vRNA8 self -folding in type A viruses. Colors indicate percentage of canonical base pairing preserved across vRNA segment 8 of type A strains. Compensating changes occur for base pairs U60/G77, C63/G74, C99/G117, C661/G814, C664/G811, U667/A808, C694/G782, A697/U779, and A703/U773. The numbering of vRNA8 is from its 5' end.

The modeled base pairs are highly conserved—average canonical base pairing is 82.6% conserved (Fig 2, and S2 Dataset). The highest base pair conservations are in domains I (98.0%) and II (91.2%). The long hairpin in region 696–780 has 92.1% average canonical base pair conservation. When mutations occur in these conserved regions they typically preserve canonical base pairing (S2 Dataset). Such structurally-neutral (consistent or compensatory) mutations suggest functional roles for these structures.

### Base pair probabilities for vRNA8 self-folding

**Fig 3:**
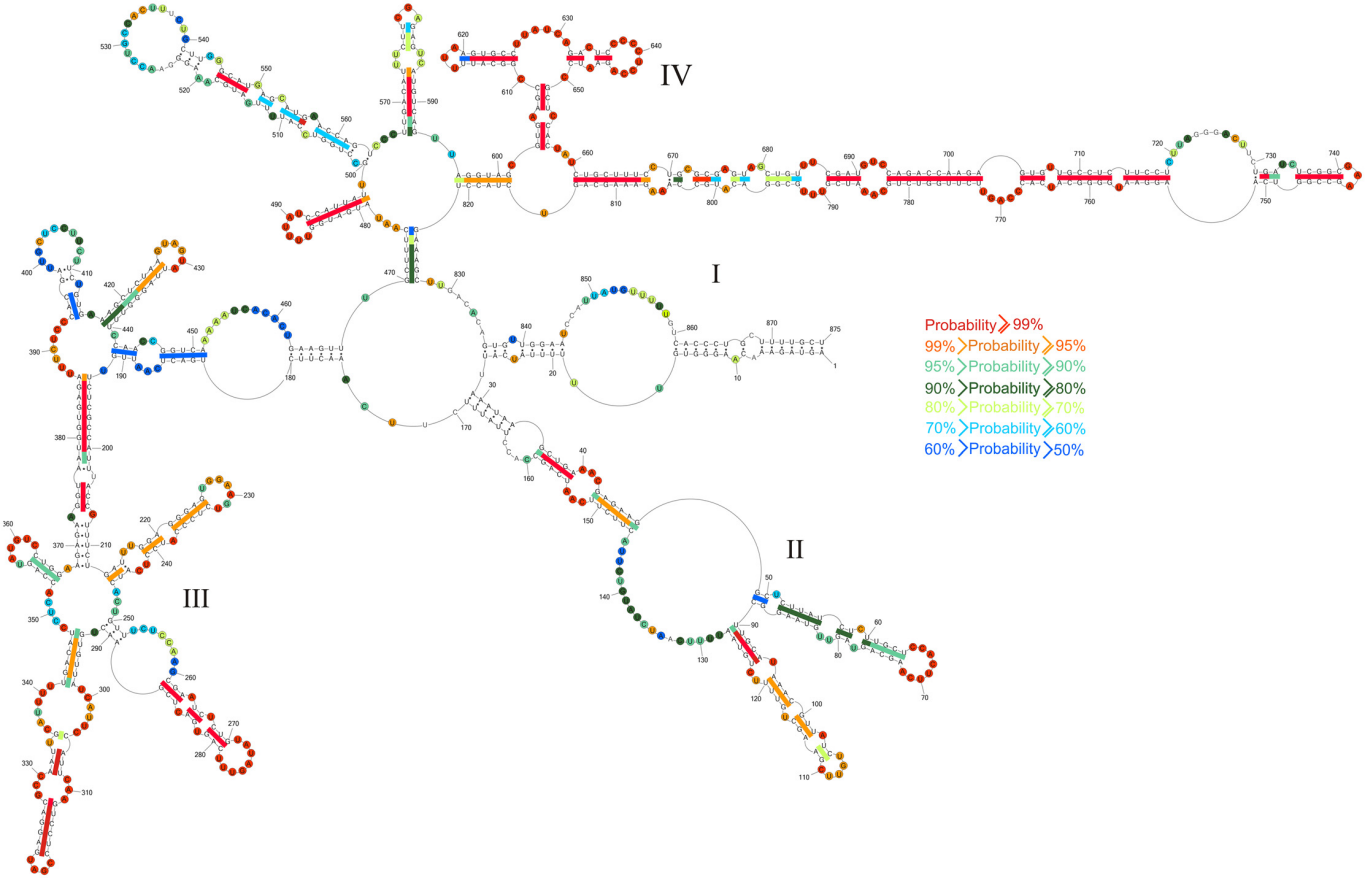
RNAstructure5.3 predicted probability of nucleotides being paired (colored lines) or single stranded (colored circles) in vRNA8 self-folding. Probability lower than 50% is not colored. The partition function calculation incorporated restraints from strong reactivity of DMS, consensus basepairs from sequence and structure analysis and SHAPE reactivities converted to pseudo-energy.

To assign probability to each base pair and unpaired nucleotide, the partition function module [69] in the RNAstructure5.3 program was used. For the partition function calculations, experimental data including sequence comparison were incorporated as restraints. Results for the vRNA8 sequence indicate that there are regions with pairs of more than 90% probability and that the most probable base pairs are generally in domain IV (Fig 3). Conversely, there are also regions of predicted low probability of particular base pairs. This includes the *panhandle* helix (1-16/861-875), which binds the heterotrimeric viral polymerase. This region is thought to be dynamic with at least two secondary structure [9, 10]. Thus the probability of particular base pairs is expected to be low.

### Microarray mapping results for vRNA8

The chemical mapping and bioinformatics provide a robust model for the secondary structure of vRNA8. Often, antisense oligonucleotides are used to inhibit RNA function. Therefore, microarray mapping [62] was used to determine vRNA8 binding sites of oligonucleotides and potentially provide additional insight into RNA folding. Oligonucleotide probes are modified to have average ΔG°_37_ of roughly -9.0 kcal/mol at 37°C (between -8.0 and -10.5 kcal/mol) for binding to unstructured pentamer or hexamer RNAs [43, 63, 64]. This largely eliminates consideration of the sequence dependence of binding when interpreting microarray results. Microarray mapping was performed under several conditions (see Materials and methods), differing in salt concentration and temperature to choose the best conditions for selective binding. The optimized condition was hybridization at 37°C in buffer A. Only strong and medium binding probes were considered.

**Fig 4:**
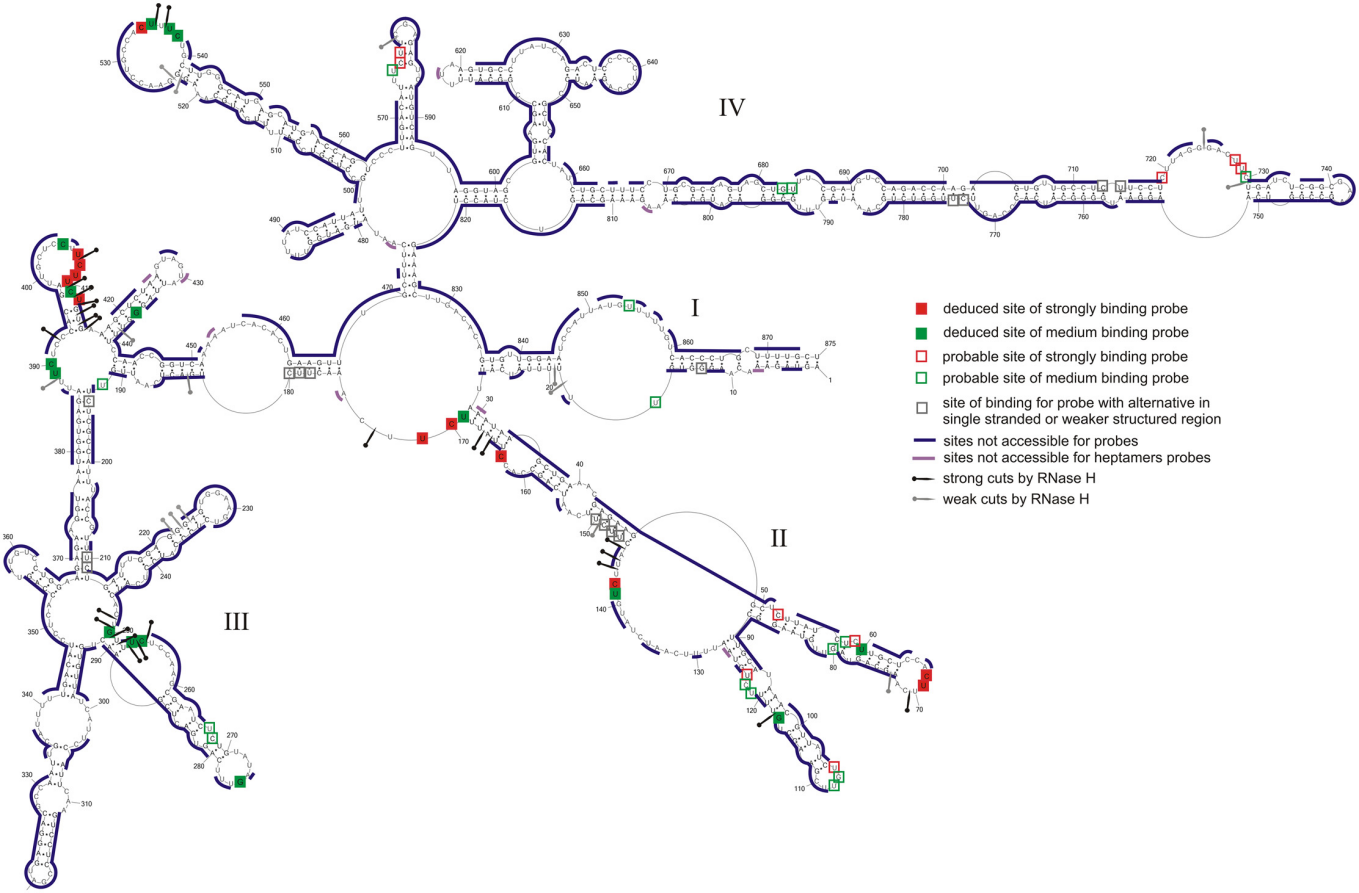
Self-folding of vRNA8 marked with regions not accesible for probes on microarray (buffer A) (see Table F in S1 File). RNase H cleavage sites for selected DNA oligonucleotides are also marked.

The isoenergetic microarray contained 454 oligonucleotides complementary to potential binding sites on segment 8 vRNA (Fig 4 and Table F in S1 File). vRNA8 hybridized to 28 probes strongly or moderately. Most probes that bind have more than one complementary potential binding site. Analysis of all possible alternative binding sites (sites within vRNA8 where oligonucleotide probes could bind via complementary and/or mismatched pairing) and their predicted free energies of hybridization identify several regions accessible for probes.

Several approaches were used to assign unambiguous strong binding sites and the relevant data for strongly binding probes are summarized in Table 1. Complete data and calculations (Table E in S1 File) allow unambiguous assignment of medium intensity binding sites (Table 2). Assigned binding sites are illustrated in Fig 1, and the logic is described below. Additional probable binding sites, i.e. those where the probe may bind to more than one site, are illustrated in Fig 4.

**Table 1:** RNase H confirmed strong binding sites in vRNA8 for microarray probes.

Confirmed binding sites ^a^	Probe sequence ^b^	Predicted ΔG°_37_ of probe/vRNA8 duplex ^c^ (kcal/mol)	Sites of strong RNase H cleavage ^d^
68/534	dDgUgg	-12.37/-12.37	70/535
69	GdDgUg	-11.17	70
142	dDgDcg	-9.58	143–145
163	dDgGug	-12.07	165–166
170/408	dDgDdg	-9.24/-11.37	172/407/410/411-415
171/409	GdDgDg	-9.74/-9.74	172/407/410/411-415
407/410	DgDdGg	-12.19/-9.61	407/410/411-415
412	dCdGdg	-9.83	410/411-415

^*a*^—binding sites are denoted by the middle nucleotide of the complementary sequence of the target;

^*b*^—nucleotides in capital letter (A, C, G, U, D) are 2’-O-methyl-RNA nucleotides, in small letter (a, c, g, u, d)—LNA nucleotides; D and d—2,6-diaminopurine (2’-O-methyl type or LNA, respectively);

^*c*^- ΔG°_37_ calculated as modified probe/RNA duplex [63, 64];

^*d*^—vRNA8 nucleotide preceding RNase H cleavage. Cleavage within 3 nucleotides of probe site was considered confirmation of probe site.

**Table 2:** Deduced medium binding sites in vRNA8 for microarray probes.

Binding sites ^a^	Probe sequence ^b^	Predicted ΔG°_37_ of probe/vRNA8 duplex ^c^ (kcal/mol)	Sites of strong RNase H cleavage ^d^	Binding to mini-vRNA8 ^e^	Deduced sites	Comments
58/*267*	dGdGdg	-10.71/-10.71	- *	S	58	Alternative site 267 not in mini vRNA8
107/ 117/ *250*/ *683*	dDcDgg	-9.15/ -9.15/ -9.15/ -9.15	117/ 248–253	M	117/ 250	
141	dGdCdg	-9.83	143–144	M	141	
121/ 169/ *210*/ *253*/ *389*/ *537*/ *575*	dGdDdg	-9.00/ -9.00/ -9.00/-9.00/ -9.00/ -11.13/ -9.00	166-167/ 250-254/ 392/ 535–536	M	169/ 253/ 389/ 537	Site 169 in mini-vRNA8 is not structurally comparable to vRNA8 region, but 169 is confirmed by RNase H cleavage
194 /*254*/ *390*	GdGdDg	-9.50/ -9.50/ -9.50	251-254/ 392	NS	254/ 390	
80/ *275*	dDcUag	-7.89/ -7.89	-	no binding	275	
*405*	dDgGdg	-12.72	407	NS	405	
122/ 180/ *211*/ *411*/ *538*/ 730	CdGdDg	-9.07/ -11.09/ -9.07/ -11.09/ -9.07/ -11.09	410-414/ 535–536	M	411/ 538	
13/ *436*	AcCcUg	-10.18/ -10.18	-	no binding	436	Site 13 is in mini-vRNA8, but site 436 is not and probe does not bind mini-vRNA8
*535*	dDdGug	-10.48	535–536	NS	535	

^*a*^—binding sites are denoted by the middle nucleotide of the complementary sequence of the target, sites in *italic* do not exist in mini-vRNA8;

^*b*^—nucleotides in capital letter (A, C, G, U, D) are 2’-O-methyl-RNA nucleotides, in small letter (a, c, g, u, d)—LNA nucleotides; D and d—2,6-diaminopurine (2’-O-methyl type or LNA, respectively);

^*c*^- ΔG°_37_ calculated as modified probe/RNA duplex [63, 64];

^*d*^—vRNA8 nucleotide preceding RNase H cleavage. Cleavage within 3 nucleotides of probe site was considered confirmation of probe site, “-“–not tested, *—site 13 was not tested for RNase H cleavage, site 436 has no strong cleavage;

*e*—symbols: S—strong binding, M—medium binding, NS—no binding and possible site not exist in mini-vRNA8.

Sites 142 and 163 are unambiguous (Table 1) because only those sites have sequences predicted to have a ΔG°_37_ favorable enough for binding their probe if the site was completely single stranded. Moreover, DNA 9-mers targeted to sites 143 and 163 produced strong RNase H cleavages at nucleotides 143–145 and 165–166 (Fig 1 and Table E in S1 File), indicating those regions are able to bind oligonucleotides.

The RNase H assay was also used to test other binding sites suggested by the microarray results (Tables 1 and 2). RNase H cleavage within three nucleotides of the target site for a strongly binding probe was considered confirmation of oligonucleotide binding to that site. Several tested sites do not undergo RNase H cleavage. However, these sites may be accessible, with less affinity for DNA, than for modified microarray probes that can form a more thermodynamically stable duplex with RNA. Therefore, negative assignment of probe binding cannot be concluded from RNase H assays. Several sites are localized more definitively by comparison with binding to a shorter construct, mini-vRNA8 (see below). In general, the deduced and probable binding sites are in good agreement with the deduced vRNA8 base pairing.

Isoenergetic probes that do not bind to vRNA8 provide additional information about structure (Fig 4). Generally, non-binding probes are in agreement with double helix regions of vRNA8. Small loops (3–4 nt) are also not accessible for oligonucleotides, which agrees with previous studies [59, 66]. Often, parts of larger hairpin loops or bulges are not accessible for probes. For example, predicted hairpin loops 225–232 and 637–644 are not accessible, which suggests strong local non-canonical or tertiary interactions (discussed below).

### Self-Folding of mini-vRNA8 on the basis of chemical mapping

The base pairing model for complete vRNA8 suggests several independently folded domains. Two regions of particular interest are those from nucleotides 1–177 and 688–875 because a mutant vRNA8 encoding only GFP protein was efficiently incorporated into virion if those nucleotides were included [45]. Therefore, the base pairing of mini-vRNA8 containing nucleotides 1–182 and 687–875 was probed.

To construct mini-vRNA8, five additional nucleotides, 5’GGAUC, were inserted to provide a restriction site of BamH1 for cloning. These nucleotides were placed in a region predicted not to change structure and where wild type coding sequence has been replaced by GFP sequence with less than a 2-fold effect on packaging [62].

**Fig 5:**
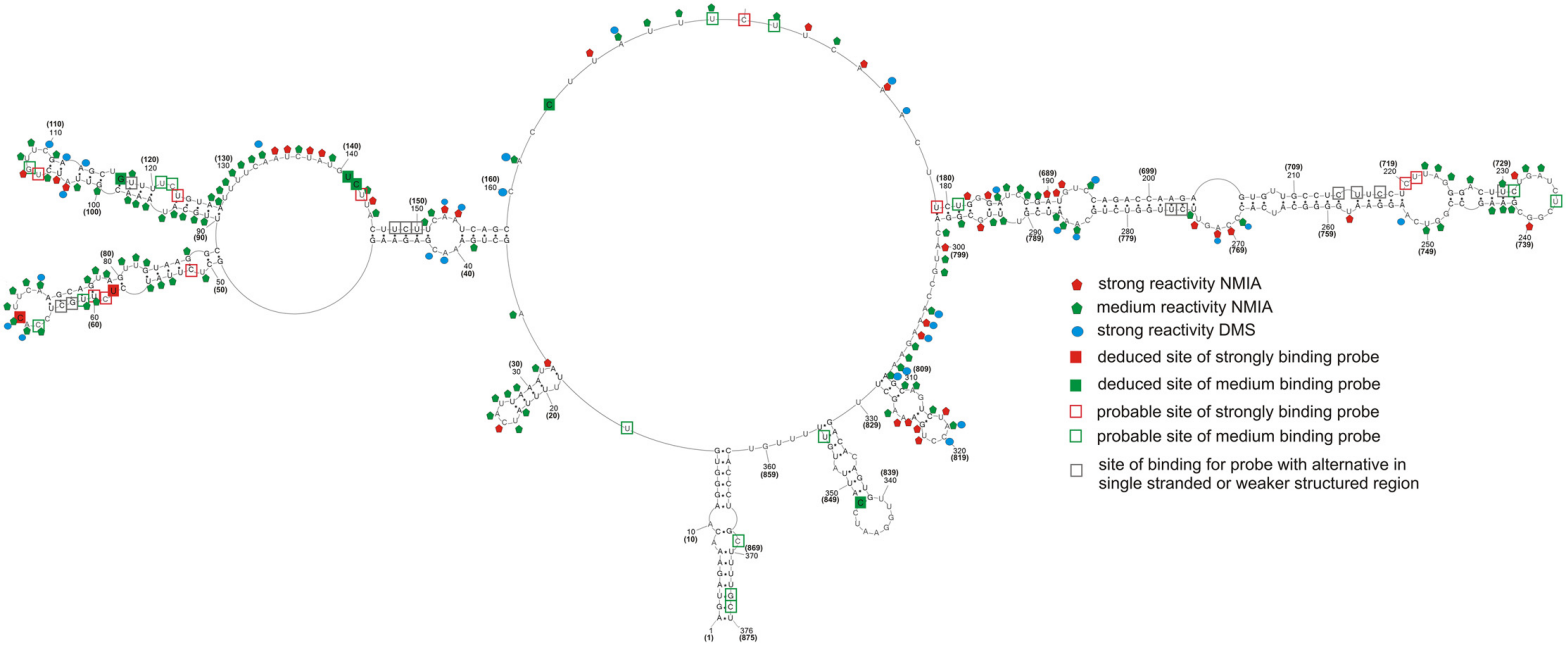
Self-folding of mini-vRNA8 predicted by RNAstructure 5.3 using as constraints: strong reactivity of DMS and SHAPE reactivities converted to pseudo-energy. Additionally there are marked results from microarray mapping in buffer A (300 mM NaCl, 5 mM MgCl_2_, 50 mM HEPES, pH 7.5) at 37°C. All symbols are the same as in Figs 1 and 4. The regions without readout of chemical mapping results are: 327–376 (826–875) (NMIA), and 322–376 (821–875) (DMS). Numbering of mini-vRNA8 is from its 5’ end and numbers in parenthesis correspond to respective nucleotides in vRNA8. Nucleotides 183–187, 5’GGAUC, were introduced for cloning (see Materials and methods). Nucleotides 1–182 and 188–376 correspond to wild type. Efficient packaging of a segment 8 encoding only GFP protein required nucleotides 1–177 and 198–376 (mini-vRNA8 nomenclature) [45].

Chemical mapping data for mini-vRNA8 were input into RNAstructure5.3 as for the entire vRNA8 (see Materials and methods). The mini-vRNA8 base pairing model (Fig 5) has three regions with secondary structure identical or similar to full length vRNA8: *panhandle* 1-16/861-875 (part of domain I), region 35–159 (part of domain II), and region 687–789 (part of domain IV). Base pairing of mini-vRNA8 differs from analogous fragments in vRNA8 only in the region connecting two preserved vRNA8 structural motifs and with three small hairpins—none of these differences affect the conserved local structures (Figs 1 and 5).

### The mini-vRNA8 results support the analysis of vRNA8

Mini-vRNA8 was more strongly modified than corresponding regions in vRNA8 (1–182 and 687–875), but similar regions are modified in both RNAs (Figs 1 and 5). Comparisons of chemical mapping between comparable regions of mini-vRNA8 and vRNA8 suggest that the region 60–113 in vRNA8 may be sequestered or involved in tertiary interactions in naked vRNA8 but not in mini-vRNA8 because more nucleotides are modified in mini-vRNA8. In contrast, the region between nucleotides 694–782 of vRNA8 is modified similarly in the two constructs, suggesting a lack of tertiary interactions. The 5’ and 3’ termini of mini-vRNA8 form a panhandle helix as in vRNA8, but the 19-27/844-836 helix in vRNA8 does not form in mini-vRNA8. This may be due to the destabilizing effect of the 2X16 nt internal loop closed by the 19-27/844-836 helix in vRNA8. In vRNA8, this internal loop may be stabilized by coaxial stacking of helix 19-27/844-836 on helix 29-34/164-169 and/or by potential tertiary helix 426-430/852-848 (Fig 1).

### Microarray mapping results for mini-vRNA8

**Fig 6:**
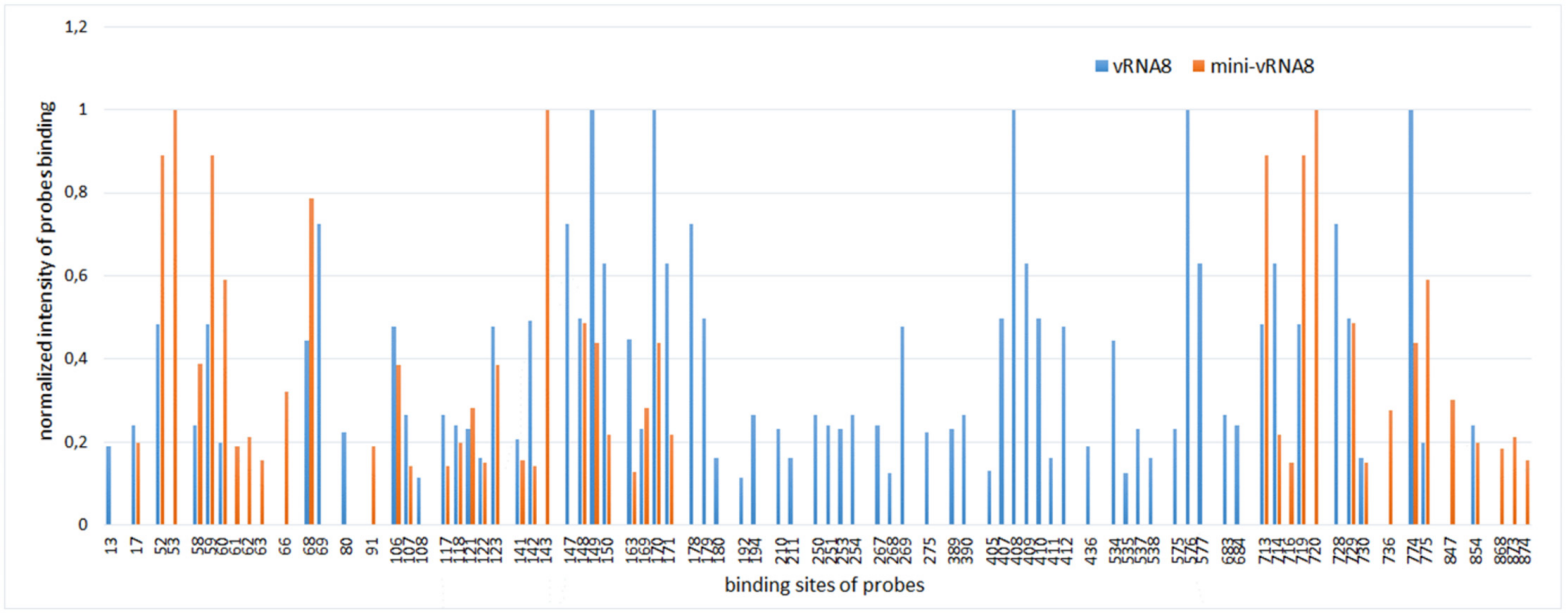
Aligned results of hybridization of vRNA8 (blue) and mini-vRNA8 (orange) to isoenergetic microarrays. All complementary sites for binding probes are shown and grouped in regions of binding.

The accessibility of mini-vRNA8 to probes in isoenergetic microarrays was compared to that of the complete vRNA8 under the same condition (37°C in buffer A) (Figs 5 and 6, Table F in S1 File). Similar regions are accessible to probes but there are several new binding sites. Binding of some probes disappears due to lack of the middle part of the complete vRNA8 sequence. Some target sites differ in relative strengths of binding. Unambiguous binding sites in the mini-vRNA8 are shown in Fig 5 and listed in Table G in S1 File. Binding of unambiguous probes is consistent with the base pairing model of mini-vRNA8 (Fig 5). Because the smaller size of mini-vRNA8 eliminates many potential alternative binding sites, unambiguous assignment of probe binding in vRNA8 can be confirmed or expanded to sites: 275, 390, 436, and 535 (Table 2 and Table G in S1 File). For example, probe 535 could bind to sites 68 and 535 in vRNA8 but does not bind to mini-vRNA8 where site 535 is absent. Therefore probe 535 binds to site 535 in vRNA8. Generally, probe binding for regions 1–173 and 687–789 are similar in vRNA8 and mini-vRNA8.

## Discussion

Influenza stores its genetic information in RNA and RNA base pairs are particularly stable thermodynamically. For example, RNA and DNA nearest neighbor interactions between Watson-Crick base pairs on average stabilize a double helix by 2.1 and 1.4 kcal/mol, respectively, at 37°C. Therefore, the equilibrium constant for forming an RNA helix will be 3-fold larger per base pair than for an equivalent DNA helix. Thus RNA has a strong propensity to self-fold and form short helixes. Here, the self-folding propensities of an influenza A segment 8 vRNA are modeled on the basis of thermodynamics, NMIA and DMS chemical mapping, and sequence comparison in order to provide insight into modules that may be important at some stage of the viral life cycle.

### Base pairing model for naked segment 8 genomic RNA of influenza A

The secondary structure model of vRNA8 (Fig 1) agrees with chemical mapping data and bioinformatics analysis of conserved influenza A canonical base pairing. The structure has four domains: I (1-27/875-836), II (29–169), III (175–468) and IV (470–827). Domains II-IV can fold independently, so all domains or even motifs in a single domain do not have to be simultaneously present at a given stage of a virus life cycle. Thus, the model in Fig 1 presents motifs that are thermodynamically stable and likely to fold. Bioinformatics analysis with RNAz 2.0 [70] of two sequences each from human, swine, and avian strains predicted self-folding in the region between nucleotides 670 and 829 [23]. The results in Fig 1 confirm stable self-folding between 670 and 804. Equivalently probable regions of self-folding were predicted by RNAz to only be present in segments 1 and 2 [23]. Perhaps segments 1, 2, and 8 have special requirements for strongly folded regions.

The modeling successfully recapitulates the one currently known functional pairing in vRNA8—annealing of the 5’ and 3’ ends to form the *panhandle* structure that is essential for viral replication [9, 10]. Evidently, this pairing is not dependent on the polymerase bringing the 5' and 3' ends together. This raises confidence in the novel structures presented here.

In general, the vRNA8 base pairing in Fig 1 is highly conserved in influenza A viruses, with average conservation of canonical base pairing of 82.6% in an alignment of over 14 000 sequences (Fig 2, and S2 Dataset). Such structurally neutral (consistent and compensatory) mutations suggest biological functions for these folds. In particular, the region 696–780 is conserved in structure and comprised of many highly preserved (>95%, Fig 2) and highly-probable (>99%, Fig 3) base pairs. This suggests that this region is likely to fold at some stage of the virus life cycle and, perhaps, have important function.

Although the biochemical analysis focused on an H5N1 strain of influenza A, the base pairs uncovered are common throughout influenza A (Fig 2). The H5N1 strain studied differs in one sense, however, from other influenza strains—there is a 15 nt deletion that brings together nt 614 and 615 in H5N1 vRNA8 (Fig 1). Interestingly, the 15 nucleotides with consensus sequence (5’CAGAGGCAAUGGUCA3’) can expand and increase the stability of the hairpin in region 611–625, while leaving the rest of the model unchanged.

Regions with low base pair probabilities may be important for function. On the basis of gel retardation experiments on protein-free RNAs, segment 8 was found to pair with segment 2, which suggests an interaction important for packaging [71]. Region 461–473 is complementary to segment 2 of Vietnam/1203/2004 (H5N1) with only one mismatch. Most segment 8 base pairs modeled in this region have low probabilities (Fig 3).

### Oligonucleotide binding does not always overlap with long runs of chemical reactivity of nucleotides

One application that benefits from determining RNA secondary structure is the design of oligonucleotide therapeutics [48, 72, 73]. Surprisingly, regions of consecutive probe binding do not always overlap with regions of consecutive nucleotides reactive to NMIA and/or DMS and vice versa (Fig 1). There are several possible reasons that probe binding and chemical modification may not always overlap. For example, tertiary interactions or weak base pairing may interfere with chemical modification [74], but probes may be able to zip open tertiary interactions and weak base pairing. Non-canonical pairs may be susceptible to chemical modification, but strong enough to reduce binding of a short oligonucleotides. These results suggest that isoenergetic microarrays can provide complementary data to biochemical probing in addition to providing lead target sites for oligonucleotide therapeutics and/or probes of function.

### Many local helixes also form in a shortened construct, mini-vRNA8

The mini-vRNA8 construct is devoid of domain III and part of domain IV (Fig 5), but contains 182 and 189 nt long 5’ and 3’ terminal regions of vRNA8 corresponding to regions 1–182 and 687–875 in Fig 1, respectively. Thus, the terminal regions include the 150 nts on each end from the coding region that give efficient packaging of a segment 8 construct encoding GFP protein [45]. The structure predicted on the basis of experimental data has mostly the same folding as in analogous regions in vRNA8 (Figs 1 and 5). Evidently, the secondary structures in these regions are self-folding domains that could be important for packaging.

### Mapping results suggest possible tertiary interactions

The single stranded regions in the vRNA8 model, especially hairpins, are generally well-supported by chemical mapping and oligonucleotide binding. In domain III, however, there are predicted single stranded regions in three hairpins and a large bulge loop (452–462) that are not reactive to chemicals. This suggests there could be alternative secondary structure for the region or possible tertiary interactions (e.g. kissing loops and pseudoknots [59, 60]) that are not captured by our model. The strongest potential tertiary interactions involve base pairing between regions: (a) 227-231/409-405 and (b) 426-430/852-848 or (b’) 400-404/431-428,426. These interactions are potentially conserved: with 88.7, 95.7 and 84.7% base pair conservation, respectively (Fig 1, Table 3). These speculative tertiary interactions may exist for naked RNA *in vitro* and explain the lack of reactivity in some regions.

**Table 3:** Possible tertiary interactions in vRNA8 structure.

Regions of interacting RNA fragments (5’→3’/3’→5’)	Sequence of fragment 1 (5’→3’)	Sequence of fragment 2 (5’→3’)	Canonical base pairs (including G-U) count of interaction for segment 8 vRNA type A (%)
(a) 227-231/409-405	GGAAG	CUUCU	88.7
(b’) 400-404/431-428,426	UGCUC	G-AGUA	84.7
(b) 426-430/852-848	GUAGU	AUUAU	95.7

There are several possible binding sites for probes in region 409–405. From experiments with complementary DNA and RNase H (Table 1), this region is clearly accessible for oligonucleotides. If the possible tertiary base pairing labeled (a) in Fig 1 and Table 3 takes place, then it is weak and probe binding apparently out-competes this tertiary interaction.

### SHAPE and DMS reactivity can provide complementary information

NMIA and DMS, respectively, interrogate ribose flexibility [75] and accessibility of Watson-Crick faces of A and C [76]. Often the reactivities do not completely overlap [57, 77, 78]. In the future, comparisons of NMIA and DMS reactivity may provide useful restraints for modeling 3D structures of RNA. The data in Fig 1 show 65 strong and 166 moderate NMIA hits and 58 strong DMS hits. There are 24 strong DMS hits that do not overlap with the strong or moderate NMIA hits. In the case of A40, A41, A153, and A154, this may reveal a local structure with adjacent AA sheared (trans Hoogsteen/Sugar-edge) base pairs. That conformation exposes the Watson-Crick faces of each A and involves base-ribose interactions [79, 80] that may reduce SHAPE reactivities. Such a correlation has been recently reported [78] for two AA pairs in the P4-P6 domain of the Tetrahymena self-splicing intron [80]. For the Bacillus subtilis RNase P specificity domain, three additional examples of A's in sheared AA or GA pairs (A56, A57, and A82) are apparent from comparing chemical mapping [77] with the crystal structure [81]. For that RNA, A55 is also only reactive with DMS and could form a sheared GA pair to complete a three purine—purine sheared pair motif [82]. In the crystal structure, however, it is part of an A55-G83-U84 base triple. As more 3D structures are determined for RNA, comparisons with reactivities of various chemical reagents may provide a foundation for improving prediction of 3D structure from secondary structure.

### Can base pairing defined in protein-free segment 8 vRNA be functional in cells?

In cells, influenza vRNA is thought to be largely, but not completely coated with NP protein that destabilizes base pairing [34, 36, 83]. RNA Watson-Crick helixes can be very stable [37] and sequence comparison is consistent with most of the base pairs determined for segment 8 (Fig 2). While unlikely that the structure in Fig 1 will completely form in cells, it would be surprising if none of the helixes present in the protein-free vRNA form at some stage of the influenza life cycle. The panhandle sequence is known to form [9, 10] and was generated by the modeling reported here. Moreover the base pairing probabilities predicted for the panhandle sequence are modest (Fig 3), which is consistent with the expected structural dynamics of this region [9, 10]. The results in Figs 1–4 suggest other regions that could be tested for possible function, e.g. regulating local speed of transcription to allow formation of pseudoknot secondary structure in mRNA, formation of tertiary interaction or binding of protein. The isoenergetic microarray results suggest regions that could be tested with antisense oligonucleotides. It might also be possible to use mutational studies, although it would be difficult to design sequences to compensate mutations while maintaining vRNA, mRNA and protein structures. Similar favorable folding is predicted for segments 1 and 2, but not 3–7 [23]. For segment 2, the most favorable folding is predicted to be 74 nts from the 3’ end similar to the 85 nts for segment 8. The results suggest that searches for possible reasons for structure in the vRNAs should be focused on segments 1, 2 and 8.

## Summary

The self-folding base pairing of an entire naked influenza RNA genomic segment *in vitro* was determined on the basis of thermodynamics and chemical mapping coupled with sequence comparison. A shorter fragment, mini-vRNA8, was also mapped. The vRNA8 self folds into many highly probable helixes. While all the structural motifs identified may not be simultaneously present in cells, the very favorable free energies for RNA folding [37, 49] would make it surprising if they never occur.

## Supporting Information

S1 Dataset vRNA8 and mini-vRNA8 chemical mapping results. (XLSX)

S2 Dataset Base pairs counts for secondary structure of vRNA8. (XLSX)

S1 File Supporting Information Tables. Primers for polymerase chain reaction using to obtain DNA template for vRNA8 and mini-vRNA8; **(Table A)**. Primers for polymerase chain reaction used to obtain mini-vRNA8 **(Table B)**. Primers for reverse transcription **(Table C)**. Heptamer probes complementary to vRNA8 **(Table D)**. RNase H cleavage of vRNA8 in the presence of selected DNA oligonucleotides **(Table E)**. Isoenergetic microarrays probes that bind strongly and moderately to vRNA8 and mini-vRNA8 and their thermodynamic properties **(Table F)**. Deduced strong and medium binding sites in mini-vRNA8 for microarray probes **(Table G)**.
(PDF)

S2 File Supporting Information Figures. vRNA8 analysis by agarose gel electrophoresis **(Fig A)**. Self-folding vRNA8 predicted by RNAstructure 5.3 without any constraints **(Fig B)**.
(PDF)
