# Supplementary material for: Self-Folding of Naked Segment 8 Genomic RNA of Influenza A Virus
Source: PLoS One. 2016 Feb 5;11(2):e0148281. doi: 10.1371/journal.pone.0148281 (PMC4743857; doi:10.1371/journal.pone.0148281)
Supplement: S1 File — Primers for polymerase chain reaction using to obtain DNA template for vRNA8 and mini-vRNA8; (Table A). Primers for polymerase chain reaction used to obtain mini-vRNA8 (Table B). Primers for reverse transcription (Table C). Heptamer probes complementary to vRNA8 (Table D). RNase H cleavage of vRNA8 in the presence of selected DNA oligonucleotides (Table E). Isoenergetic microarrays probes that bind strongly and moderately to vRNA8 and mini-vRNA8 and their thermodynamic properties (Table F). Deduced strong and medium binding sites in mini-vRNA8 for microarray probes (Table G). (PDF) [file pone.0148281.s003.pdf]

## S1 File. Supporting Information Tables.

**Table A. Primers for polymerase chain reaction using to obtain DNA template for vRNA8 and mini-vRNA8.**

| Name | Length (nt) | Sequence 5’→3’ [a] |
|---|---|---|
| Rev | 30 | AGCAAAAGCAGGGTGACAAAAACATAATGG |
| For | 61 | AAAAATAATACGACTCACTATAGGGAGTAGAAACAAGGGTGTTTTTTATCATTAAATAAGC |

[a] The underlined nucleotides residues are the polymerase T7 promoter.

**Table B. Primers for polymerase chain reaction using to obtain mini-vRNA8.**

| | Length (nt) | Sequence 5’→3’ [a] |
|---|---|---|
| 1 | 36 | AAGAATTCAGTAGAAACAAGGGTGTTTTTTATCATT |
| 2 | 33 | AAGGATCCCAGAAGTTTGAAGAAATAAGGTGGC |
| 3 | 30 | AAGGATCCGATGTCCAGACCAAGAGTGTTG |
| 4 | 25 | AACTGCAGAGCAAAAGCAGGGTGAC |

[a] The underlined nucleotides residues are restriction sites: 1 - EcoR1, 2, 3 - BamH1, 4 - Pst1.

**Table C. Primers for reverse transcription.**

| Name | Length (nt) | Complementary region in segment 8 RNA | Sequence 5’→3’ [a] |
|---|---|---|---|
| Pr1 | 23 | 847-869 | AGCaGGgTGaCAAaAACaTAATG |
| Pr2 | 22 | 719-740 | GCCgAGaTCaGAAgTCCCTaAG |
| Pr3 | 23 | 572-591 | CTGACATGACTCTCGAAGAAATG |
| Pr4 | 26 | 287-312 | CTTGAATGGAATGATAACACAGTTCG |
| Pr5 | 22 | 429-450 | GACCGGTTGGAAACCCTAATAC |
| Pr6 | 22 | 142-163 | GGTGGCTGATTGAAGAAGTAAG |

[a] DNA primers 1-6 were used to vRNA8; primers 1, 2, 6 to mini-vRNA8; in small letter (a, g) - LNA nucleotides. Each primer was labeled with 6-FAM at 5’ end.

**Table D**. **Heptamer probes complementary to vRNA8.**

| Complementary binding sites | Sequence and modifications of probes [a] 5'→3' |
|---|---|
| 7 | UgUuUcUPy |
| 30 | UdUuUdAPy |
| 126 | AdUuAcAPy |
| 174 | GuUuGdAPy |
| 424 | AcUuAgAPy |
| 430 | AdUdCuAPy |
| 454 | GdUuUuUPy |
| 476 | UdUuGdAPy |
| 618 | CuUdAdAPy |
| 806 | UuCuUuGPy |
| | |

[a] nucleotides in capital letter (A, C, G, U, D) are 2'-O-methyl-RNA nucleotides, in small letter (a, c, g, u, d) - LNA nucleotides; D and d - 2,6 –diaminopurine (2'-O-methyl type or LNA, respectively). Py – pyrene (56-57).

**Table E. RNase H cleavage of vRNA8 in the presence of selected DNA oligonucleotides.**

| Binding sites for vRNA8 | Sequence of DNA oligonucleotide [a] | Predicted $\Delta G°_{37}$ for DNA/RNA duplex [b] (kcal/mol) | Predicted $\Delta G°_{37}$ of duplex calculated as RNA/RNA[c] (kcal/mol) | Predicted $\Delta G°_{37}$ of mismatched duplex calculated as RNA/RNA [c] (kcal/mol) | RNase H cleavage site [d] |
|---|---|---|---|---|---|
| 68 (534) | AAGTGG | -3.44 | -6.3 (534)<br>-6.2 (68) | | 70 (s)<br>535 (s) |
| 194 (254, 390) | GAGAAG | -2.20 | -5.7 (194)<br>-5.0 (390)<br>-5.0 (254) | -4.8 (148/149)<br>-4.6 (729)<br>-4.6 (179)<br>-4.6 (407/408)<br>-4.3 (57/58)<br>-4.3 266/267 | 248-254 (s)<br>388 (w)<br>392 (s)<br>407 (s)<br>410 (s) |
| 17 (118, 251, 684, 854) | AAACAG | -1.47 | -4.2 (251)<br>-3.9 (684)<br>-3.9 (118)<br>-1.8 (17)<br>-1.8 (854) | -3.5 (107) | 18-19 (w)<br>117 (s)<br>249 (s) |
| 108 (192) | GAACAG | -2.63 | -7.4 (108)<br>-5.2 (192) | -4.6 (250/251)<br>-4.3 (683/684)<br>-4.3 (117/118) | 249-250 (s) |
| 143 | AGTAAGACA | -6.43 | -10.6 | -6.1 (61/62)<br>-6.1 (781/782)<br>-5.9 (707)<br>-5.5 (488)<br>-5.4 (91)<br>-5.4 (816/817) | 143-145 (s) |
| 163 | ATAAGGTGG | -7.10 | -12.0 | -7.3 (818)<br>-7.3 (534)<br>-7.0 (626)<br>-6.5 (67/68)<br>-6.3 (197/198)<br>-6.3 (352)<br>-6.0 (766/767) | 165-166 (s) |

| | | | | | |
|---|---|---|---|---|---|
| 170 | TGAAGAAAT | -3.83 | -8.7 | -8.1 (575)<br>-7.5 (150)<br>-6.3 (408/409)<br>-5.0 (714)<br>-4.8 (70)<br>-4.5 (253) | 172 (s)<br>251 (s)<br>253 (s)<br>149 (w)<br>73 (w) |
| 408 | AGAAGAAGG | -5.72 | -12.4 | -10 (149)<br>-7.2 (170)<br>-7.2 (576)<br>-6.9 (713) | 411-415 (s) |
| 436 | AAACCCTAA | -7.25 | -10.2 | -7.9 (13)<br>-7.5 (722/723)<br>-7.0 (759)<br>-6.3 (221/222)<br>-6.2 (522/523) | 438 (w)<br>221-223 (w)<br>522-523 (w)<br>724 (w) |
| 535 | AGAAAGTGG | -6.36 | -12.3 | -8.5 (657)<br>-7.6 (664/665)<br>-7.5 (68)<br>-6.7 (472) | 535-536 (s) |
| 576 | CGAAGAAAT | -4.43 | -9.9 | -8.0 (169/170)<br>-6.8 (149/150)<br>-6.5 (408/409)<br>-5.3 (714) | 578 (w)<br>172 (w) |
| 721 | CCCTAAGAG | -8.78 | -14.8 | -7.8 425 | |
| 729 | TCAGAAGTC | -7.65 | -12.6 | -10.3 (179/180)<br>-6.9 (410/411)<br>-6.6 (211) | 730 (w)<br>182 (w) |

*a* – Sequences of DNA 6-mers were the same as probe for certain site, sequences of DNA 9-mer were specific for designated site; *b* - calculated for DNA/RNA duplex in 300 mM NaCl, as in experiment (http://ozone3.chem.wayne.edu); *c* - calculated in RNAstructure5.3 program as RNA/RNA duplex for standard condition (1 M NaCl) to show the difference in $\Delta G^\circ_{37}$ between complementary and predicted mismatched duplexes, in parenthesis - site of possible DNA oligonucleotide binding, denoted by the middle nucleotide of the complementary RNA region (or two nucleotides for duplex with an even number of nucleotides; *d* – nucleotides preceding RNase H cleavage site; s-strong cut, w-weak cut.

**Table F. Isoenergetic microarrays probes that bind strongly and moderately to vRNA8 and mini-vRNA8 and their thermodynamic properties[a].**

| Probe name [b] | Binding sites for vRNA8 [c] | Probe sequence [d] | Strength of probe binding [e] | | $\Delta G^\circ_{37}$ of duplex for complementary binding site [f] (kcal/mol) | Nucleotide of RNA target complementary to 3'g of hexamer probe | $\Delta G^\circ_{37}$ of duplex for possible mismatched sites [f,g] (kcal/mol) |
|---|---|---|---|---|---|---|---|
| | | | vRNA8 | mini-vRNA8 | | | |
| 1p | 149 (170, *408*, *576*, 774) | dDgDdg | 1.000 (S) | 0.4391 (S) | -4.4 (-11.37) 149<br>-4.2 (-11.37) 408<br>-2.7 (-9.24) 774<br>-2.3 (-9.24) 170<br>-2.3 (-9.24) 576 | 149C<br>170U<br>408C<br>576U<br>774G | -4.5 (-9.34) **179**<br>-4.5 (-9.34) **729**<br>-4.1 (-6.0) **69/70** |
| 2p | 69 (147, 178, 728) | GdDgUg | 0.7241 (S) | - | -7.6 (-11.17) 69<br>-5.4 (-9.23) 728<br>-5.4 (-10.03) 147<br>-5.3 (-10.03) 178 | 69C<br>147U<br>178A<br>728G | -4.4 (-9.45) **534/535** |
| 3p | 150 (171, *409*, *577*, 714) | GdDgDg | 0.6299 (S) | 0.2180 (M) | -6.9 (-11.76) 714<br>-5.9 (-9.74) 171<br>-5.9 (-9.74) 150<br>-5.9 (-9.74) 577<br>-5.4 (-9.74) 409 | 150U<br>171U<br>409U<br>577U<br>714C | -4.2 (-8.53) **52**<br>-4.2 (-8.53) **59**<br>-4.1 (-5.47) **69**/70 |
| 4p | 148 (179, *407*, *410*, 729) | DgDdGg | 0.4967 (S) | 0.4877 (S) | -6.4 (-12.19) 407<br>-5.2 (-9.61) 179<br>-5.2 (-9.61) 729<br>-5.1 (-9.61) 410<br>-4.6 (-9.61) 148 | 148A<br>179A<br>407C<br>410U<br>729A | -4.7 (-10.08) **536/537**<br>-4.6 (-4.77) **69/70**<br>-4.5 (-4.77) **171/172**<br>-4.5 (-4.77) **577/578** |
| 5p | 142 | dDgDcg | 0.4913 (S) | 0.1412 (M) | -5.4 (-9.58) 142 | 142U | |
| 6p | 52 (59, 713, 719) | dDgDgg | 0.4837 (S) | 0.8894 (S) | -7.0 (-12.96) 719<br>-6.5 (-12.96) 713<br>-5.1 (-10.03) 59<br>-5.0 (-10.03) 52 | 52G<br>59U<br>713C<br>719C | -4.6 (-10.03) **267/268**<br>-4.2 (-8.52) **729**<br>-4.2 (-8.52) **179**<br>4.1 (-10.55) **148/149** |
| 7p | 106 (123, *269*, *412*) | dCdGdg | 0.4772 (S) | 0.3867 (S) | -7.1 (-11.96) 269<br>-5.4 (-9.83) 123<br>-5.4 (-9.83) 412<br>-4.6 (-9.83) 106 | 106A<br>123U<br>269C<br>412U | -4.1 (-5.96) **538/539**<br>-4.1 (-5.96) **180**/181<br>-4.1 (-5.96) **730/731**<br>-4.1 (-5.96) **211**/212 |
| 8p | 163 | dDgGug | 0.4470 (S) | 0.1264 (M) | -7.0 (-12.07) 163 | 163C | |
| 9p | 68 (*534*) | dDgUgg | 0.4449 (S) | 0.787 (S) | -6.3 (-12.37) 534<br>-6.2 (-12.37) 68 | 68C<br>534C | |

| | | | | | | | |
|---|---|---|---|---|---|---|---|
| 10p | 107 (117, *250*, *683*) | dDcDgg | 0.2661 (M) | 0.1429 (M) | -4.4 (-9.15) 250<br>-4.4 (-9.15) 107<br>-4.2 (-9.15) 683<br>-4.2 (-9.15) 117 | 107U<br>117G<br>250A<br>683G | -4.3 (-5.62) **412/413**<br>-4.2 (-5.62) **269**/270<br>-4.2 (-5.62) **123/124** |
| 11p | 194 (*254*, *390*) | GdGdDg | 0.2659 (M) | - | -5.7 (-9.5) 194<br>-5.0 (-9.5) 390<br>-5.0 (-9.5) 254 | 194G<br>254U<br>390U | -4.8 (-9.8) **148/149**<br>-4.6 (-8.29) **729**<br>-4.6 (-8.29) **179**<br>-4.6 (-9.8) **407/408**<br>-4.3(-6.39) **57/58**<br>-4.3 (-6.39) **266/267** |
| 12p | 58 (*267*) | dGdGdg | 0.2393 (M) | 0.3886 (S) | -5.4 (-10.71) 267<br>-4.8 (-10.71) 58 | 58A<br>267A | -5.1 (-10.38) **536/537**<br>-5.0 (-6.39) **194**/195<br>-4.8 (-8.3) **719**<br>-4.3 (-7.89) **390/391**<br>-4.4 (-6.39) **254/255** |
| 13p | 17 (118, *251*, *684*, 854) | dDdcDg | 0.2387 (M) | 0.1989 (M) | -4.2 (-10.49) 251<br>-3.9 (-10.49) 684<br>-3.9 (-10.49) 118<br>-1.8 (-8.47) 17<br>-1.8 (-8.47) 854 | 17G<br>118C<br>251C<br>684C<br>854A | -3.5 (-8.46) **107** |
| 14p | 121 (169, *210*, *253*, *389*, *537*, *575*) | dGdDdg | 0.2302 (M) | 0.2805 (M) | -5.0 (-11.13) 537<br>-2.9 (-9.00) 121<br>-2.7 (-9.00) 210<br>-2.1 (-9.00) 253<br>-2.1 (-9.00) 169<br>-2.1 (-9.00) 389<br>-2.1 (-9.00) 575 | 121U<br>169A<br>210A<br>253G<br>389A<br>537C<br>575A | |
| 15p | 80 (*275*) | dDcUag | 0.2241 (M) | - | -2.9 (-7.89) 80<br>-2.3 (-7.89) 275 | 80G<br>275A | |
| 16p | 141 | dGdCdg | 0.2072 (M) | 0.1543 (M) | -4.6 (-9.83) 141 | 141A | -4.3 (-9.50) **250/251**<br>-4.0 (-9.50) **117/118**<br>-4.0 (-9.50) **683/684** |
| 17p | 60 (775) | cDdGdg | 0.1966 (M) | 0.5904 (S) | -6.0 (-11.62) 60<br>-5.2 (-9.49) 775 | 60C<br>775U | -4.0 (-9.10) **719** |
| 18p | 13 (*436*) | AcCcUg | 0.1905 (M) | - | -7.6 (-10.18) 13<br>-7.1 (-10.18) 436 | 13A<br>436U | |

| | | | | | | | |
|---|---|---|---|---|---|---|---|
| 19p | 122 (180, *211*, *411*, *538*, 730) | CdGdDg | 0.1614 (M) | 0.1508 (M) | -7.1 (-11.09) 180<br>-7.1 (-11.09) 730<br>-6.2 (-11.09) 411<br>-5.0 (-9.07) 211<br>-4.5 (-9.07) 122<br>-4.3 (-9.07) 538 | 122U<br>180C<br>211U<br>411C<br>538U<br>730C | -4.0 (-8.29) **148** |
| 20p | *405* | dDgGdg | 0.1302 (M) | - | -6.5 (-12.72) 405 | 405C | -4.0 (-8.47) **729**<br>-4.0 (-8.47) **179** |
| 21p | *535* | dDdGug | 0.1254 (M) | - | -4.0 (-10.48) 535 | 535C | -3,7 (-8.45) **68** |
| 22p | *268* | CdGdGg | 0.1239 (M) | - | -6.7 (-9.86) 268 | 268U | -6.8 (-10.33) **179/180**<br>-6.8 (-10.33) **729/730**<br>-5.9 (-10.33) **410/411**<br>-4.7 (-7.08) **211**<br>-4.2 (-7.08) **122**<br>-4.1 (-5.56) **58/59**<br>-4.0 (-5.96) **538**<br>-3.7 (-7.53) **148**<br>-3.6 (-5.1) **163/164** |
| 23p | 108 (*192*) | GDdCdg | 0.1126 (M) | - | -7.4 (-10.64) 108<br>-5.2 (-8.51) 192 | 108C<br>192U | -4.6 (-8.92) **250/251**<br>-4.3 (-8.92) **683/684**<br>-4.3 (-8.92) **117/118** |
| 24p | 53 (143, 720) | uDdGdg | - | 1.000 (S) | -5.3 (-10.91) 720<br>-4.5 (-10.91) 53<br>-2.9 (-8.78) 143 | 53C<br>143G | -4.5 (-10.91) **59/60** |
| 25p | 61 (91) | GcDdGg | - | 0.1904 (M) | -7.5 (-9.87) 91<br>-7.1 (-9.87) 61 | 61U<br>91G | |
| 26p | 62 (873) | DgCdDg | - | 0.2114 (M) | -6.2 (-11.89) 62<br>-4.5 (-9.87) 873 | 62C<br>873U | |
| 27p | 63 (874) | GdGCdg | - | 0.1555 (M) | -7.2 (-10.44) 63<br>-6.2 (-10.44) 874 | 63U<br>874U | -7.4 (-10.17) **107**/108 |
| 28p | 66 | GUgGdg | - | 0.3200 (M) | -9.4 (-12.52) 66 | 66C | -5.0 (-7.27) **847** |

| | | | | | | | |
|---|---|---|---|---|---|---|---|
| 29p | 716 | DgGdDg | - | 0.1506 (M) | -6.4 (-12.19) 716 | 716C | -4.1 (-9.78) **148/149**<br>-4.1 (-7.48) **729** |
| 30p | 736 | CGdGdg | - | 0.2762 (M) | -6.8 (-10.24) 736 | 736A | -5.7 (-12.37) **59/60**<br>-4.4 (-9.10) **719**<br>-4.3 (-6.17) **52** |
| 31p | 847 | dUgGdg | - | 0.3015 (M) | -4.7 (-10.46) 847 | 847A | |
| 32p | 868 | dDgCdg | - | 0.1824 (M) | -6.4 (-12.42) 868 | 868C | -4.2 (-9.11) **62**<br>-4.2 (-6.76) 873/**874**<br>-3.9 (-7.59) **117/118**<br>-3.5 (-7.69) **107**<br>-3.2 (-5.39) **123**/124 |
| 33p | 181* | CCdGdg | - | 0.1133 (M) | -7.6 (-10.27) 181* | 181U | -4.5 (-5.96) **730**/731<br>-3.8 (-7.46) **122**<br>-3.8 (-5.96) 105/**106** |

*a* – All sites mapped by microarray mapping are marked on Figure S3; binding sites of probes are denoted by the middle nucleotide of the complementary RNA region (or two nucleotides for probes with an even number of nucleotides; *b* – probes 1p-23p bind vRNA8 and part of them mini-vRNA8, probes 24p-33p bind only mini-vRNA8; *c* - in parenthesis are other fully complementary binding sites for the probe; sites in *italic* do not exist in mini-vRNA8; *d* - nucleotides in capital letter (A, C, G, U, D) are 2'-O-methyl-RNA nucleotides, in small letter (a, c, g, u, d) are LNA nucleotides, D and d are 2,6 –diaminopurine (2'-O-methyl type or LNA, respectively); *e* - value: 0.33 ≤ strong (S), 0.11 ≤ medium (M) <0.33 and no binding (-) < 0.11. Condition: buffer A (300 mM NaCl, 5 mM $MgCl_2$, 50 mM HEPES, pH 7.5), 37°C; *f* - calculated in RNAstructure program as RNA/RNA duplex and (in parenthesis) calculated considered modification of probe as modified probe/RNA duplex [1,2] the last number is the site of binding for which calculation was done; *g* - bolded are complementary sites of probe which binds strongly or moderatly; *- only mini-vRNA8 site.

**Table G. Deduced strong and medium binding sites in mini-vRNA8 for microarray probes.**

| Probable binding sites [a] | Probe sequence [b] | Predicted $\Delta G°_{37}$ of probe/vRNA8 duplex [c] (kcal/mol) | Sites of strong RNase H cleavage in vRNA8 [d] | Strength of binding [e] | Deduced sites | Comments |
|---|---|---|---|---|---|---|
| 58 | dGdGdg | -10.71 | - | M | 58 | No alternative sites |
| 68 | dDgUgg | -12.37 | 70 | S | 68 | No alternative sites |
| 107 (117) | dDcDgg | -9.15/ -9.15 | 117 | M | 117 | Strong RNase H cleavage at 117 in vRNA8, which is in the region of simiar folding in mini-vRNA8 and vRNA8 |
| 141 | dGdCdg | -9.83 | 143-144 | M | 141 | Strong RNase H cleavage at 143-144 in vRNA8, which is in the region of simiar folding in mini-vRNA8 and vRNA8 |
| 142 | dDgDcg | -9.58 | 143-144 | S | 142 | No alternative sites |
| 163 | dDgGug | -12.07 | 166-167 | M | 163 | No alternative sites |
| 847 | dUgGdg | -10.46 | - | M | 847 | No alternative sites |

*a* - binding sites are denoted by the middle nucleotide of the complementary sequence of the target; *b* - nucleotides in capital letter (A, C, G, U, D) are 2’-O-methyl-RNA nucleotides, in small letter (a, c, g, u, d) - LNA nucleotides; D and d - 2,6-diaminopurine (2’-O-methyl type or LNA, respectively); *c*- $\Delta G°_{37}$ calculated as modified probe/RNA duplex [1,2]; *d* - vRNA8 nucleotide preceding RNase H cleavage. Cleavage within 3 nucleotides of probe site was considered confirmation of probe site, “-“ – not tested; *e* – symbols: S – strong binding, M – medium binding.

## References


1. Pasternak A, Kierzek E, Pasternak K, Fratczak A, Turner DH. and Kierzek R. The thermodynamics of 3'-terminal pyrene and guanosine for the design of isoenergetic 2'-O-methyl-RNA-LNA chimeric oligonucleotide probes of RNA structure. Biochemistry 2008;47: 1249-1258.
2. Kierzek E, Ciesielska A, Pasternak K, Mathews DH, Turner DH and Kierzek R. The influence of locked nucleic acid residues on the thermodynamic properties of 2'-O-methyl RNA/RNA heteroduplexes. Nucleic Acids Res. 2005; 33: 5082-5093.
