# Supplementary material for: Self-Folding of Naked Segment 8 Genomic RNA of Influenza A Virus
Source: PLoS One. 2016 Feb 5;11(2):e0148281. doi: 10.1371/journal.pone.0148281 (PMC4743857; doi:10.1371/journal.pone.0148281)
Supplement: S2 File — vRNA8 analysis by agarose gel electrophoresis (Fig A). Self-folding vRNA8 predicted by RNAstructure 5.3 without any constraints (Fig B). (PDF) [file pone.0148281.s004.pdf]

S2 File. Supporting Information Figures.

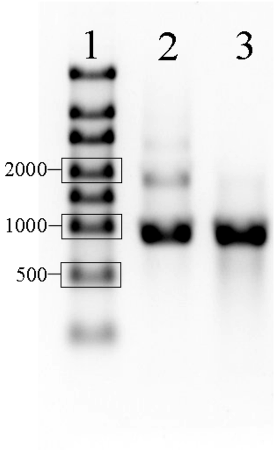

Fig A. vRNA8 analysis by agarose gel electrophoresis. 1) RNA Ladder - RiboRuler High Range RNA Ladder – bands in boxes with numbers refer to RNA length; 2) vRNA8 without folding – the homodimeru is present; 3) vRNA8 after folding in buffer (300mM NaCl, 5mM MgCl2, 50mM HEPES pH 7.5) (see Materials and methods).

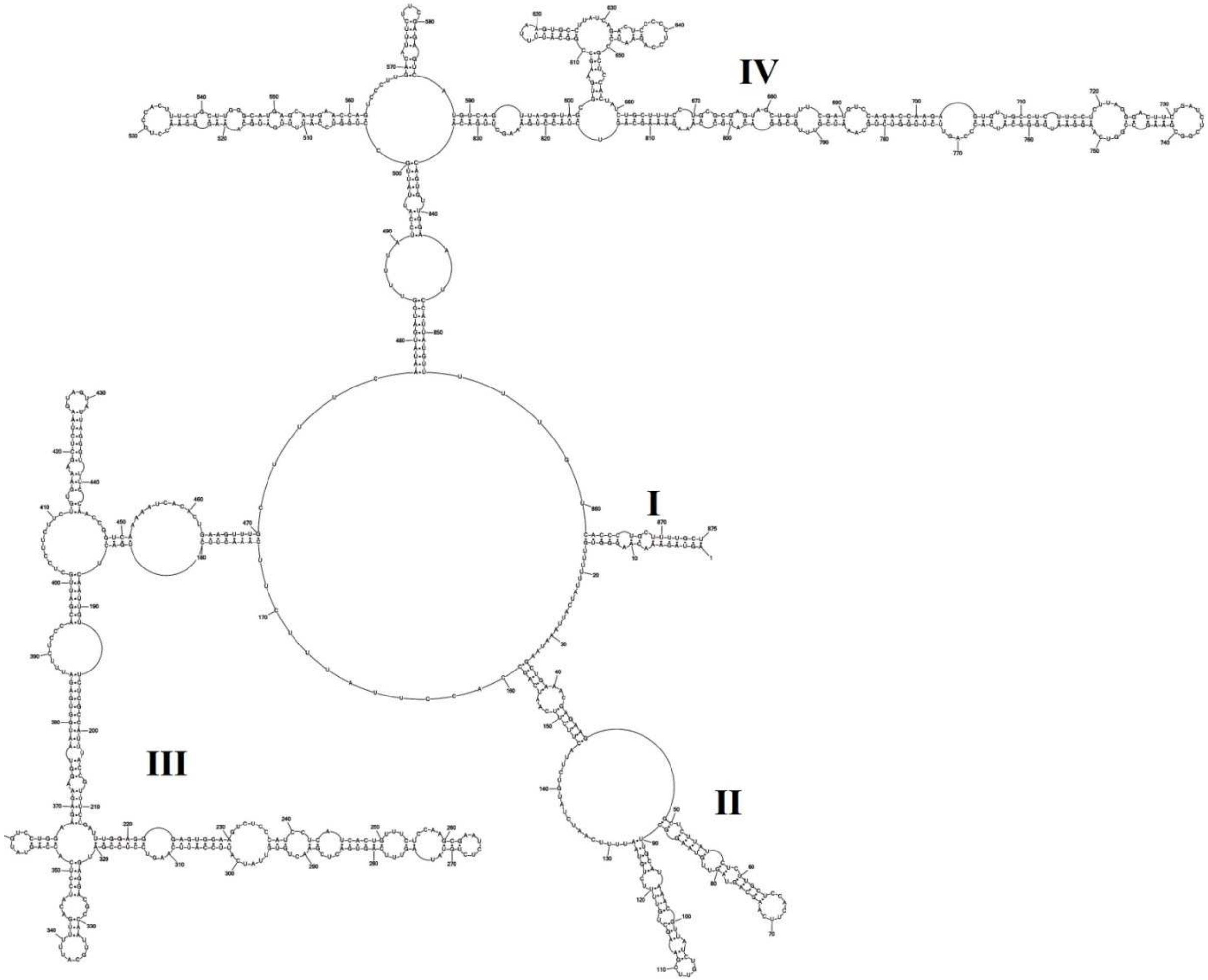

Fig B. Self-folding vRNA8 predicted by RNAstructure 5.3 without any constraints. The numbering of vRNA8 is from its 5' end.
